# Diabetic Retinopathy: Mitochondria Caught in a Muddle of Homocysteine

**DOI:** 10.3390/jcm9093019

**Published:** 2020-09-19

**Authors:** Renu A. Kowluru

**Affiliations:** Department of Ophthalmology, Visual and Anatomical Sciences, Wayne State University, Detroit, MI 48201, USA; rkowluru@med.wayne.edu; Tel.: +1-313-993-6714

**Keywords:** diabetic retinopathy, epigenetics, homocysteine, mitochondria

## Abstract

Diabetic retinopathy is one of the most feared complications of diabetes. In addition to the severity of hyperglycemia, systemic factors also play an important role in its development. Another risk factor in the development of diabetic retinopathy is elevated levels of homocysteine, a non-protein amino acid, and hyperglycemia and homocysteine are shown to produce synergistic detrimental effects on the vasculature. Hyperhomocysteinemia is associated with increased oxidative stress, and in the pathogenesis of diabetic retinopathy, oxidative stress-mitochondrial dysfunction precedes the development of histopathology characteristic of diabetic retinopathy. Furthermore, homocysteine biosynthesis from methionine forms S-adenosyl methionine (SAM), and SAM is a co-substrate of DNA methylation. In diabetes, DNA methylation machinery is activated, and mitochondrial DNA (mtDNA) and several genes associated with mitochondrial homeostasis undergo epigenetic modifications. Consequently, high homocysteine, by further affecting methylation of mtDNA and that of genes associated with mtDNA damage and biogenesis, does not give any break to the already damaged mitochondria, and the vicious cycle of free radicals continues. Thus, supplementation of sensible glycemic control with therapies targeting hyperhomocysteinemia could be valuable for diabetic patients to prevent/slow down the development of this sight-threatening disease.

## 1. Introduction

Diabetes, a life-long disease, is now considered as one of the fastest growing health challenges of the 21st century with nearly 463 million people between the age of 21–79 having diabetes (Diabetes atlas, 9th edition, 2019, International Diabetes Federation). Diabetes affects most of the organs of the body resulting in microvascular and macrovascular complications; diabetic patients have a two times higher risk of cardiovascular diseases, a ten times higher risk of end-stage renal disease, and are over two times more likely to develop cataracts and open-angle glaucoma [1]. One of the major microvascular complications of diabetes is retinopathy. This progressive disease eventually affects virtually all patients with diabetes and is the leading cause of blindness in working-aged adults [2]. Worldwide, 127 million people had diabetic retinopathy in 2010, and this number is expected to reach over 190 million in 2030 [3]. The retina, a densely vascular neuronal tissue that converts light signals into electric signals to transmits them to the brain is damaged by sustained circulating glucose, and its vasculature begins to leak and neuronal cells die [4]. Focal laser treatment to stop/slow the leakage, pan-retinal coagulation to shrink the abnormal vasculature, and vitrectomy to remove blood from the vitreous have been routinely used in the clinic, but these treatments carry many unwanted effects including destruction of other parts of the retina. Anti-vascular endothelial growth factor therapies are also now routinely used to stop the growth of new blood vessels, but their effects last only for a short duration (4–6 weeks) and require frequent visits to the ophthalmologist for this life-long disease [5]. Good glycemic control prevents/retards the development of diabetic retinopathy [4], but maintenance of such glycemic control becomes difficult, or impossible, over a long duration, thus, adjunct therapies are desperately needed to relieve patients from the fear of losing their vision.

The main initiator of diabetic complications is considered to be sustained hyperglycemia; however, in addition to non-modifiable factors (e.g., duration of diabetes), other modifiable ones are also associated with its development including hyperlipidemia, obesity, and hypertension. Consequently, lipid-lowering agents and the maintenance of good blood pressure have retarded the progression of diabetic retinopathy [6]. Diabetic patients may also have moderately elevated levels of a non-protein amino acid, homocysteine (~30 μmol/L) [7]; plasma concentration of homocysteine ranges from 3 to 15 μmol/L in healthy individuals, patients with levels between 16 and 100 μmol/L are considered mild-to-moderate hyperhomocysteinemic, and those with above 100 μmol/L are severely hyperhomocysteinemic [8,9]. Homocysteine-associated toxicity is linked with the diseases that include involvement of nearly all organ systems including the heart, kidney, and eye [10]. High homocysteine is associated with many retinal disorders including retinal vein occlusion [11], endothelial cell dysfunction [12], and disruption of the retinal pigment epithelium [13]. Homocysteine and diabetes have apparent synergistic detrimental vascular effects [14], and experimental models have shown that supplementation with homocysteine in hyperglycemic conditions exacerbates metabolic abnormalities associated with the development of diabetic retinopathy [15]. Diabetic retinopathy is a progressive disease, and the differences in the duration of diabetes in various studies and the methods used to evaluate the severity of the disease have produced inconsistent results in establishing association between homocysteine and diabetic retinopathy. However, despite some inconsistencies, many clinical studies have documented a significant relationship between hyperhomocysteinemia and retinopathy in type 1 and type 2 diabetic patients. For example, type I diabetic patients without any complications present lower homocysteine levels compared to diabetic patients with retinopathy [16,17]. In addition, several meta-analyses have also shown an association—type 1 diabetic patients with retinopathy have elevated homocysteine levels, compared to diabetic patients without any complications [18]. A meta-analysis of 31 studies with 6394 participants indicates hyperhomocysteinemia as one of the risk factors of diabetic retinopathy, especially proliferative diabetic retinopathy [19], and another analysis of 11 studies with 2184 diabetic patients shows an association between homocysteine and increased risk of diabetic retinopathy [20]. Type 2 diabetic patients with proliferative retinopathy also have significantly higher homocysteine levels (~18 µmol/L) compared to the control healthy subjects (~7.8 µmol/L) [8] and show an association between increase in plasma homocysteine and increased risk of developing diabetic macular edema [21]. Furthermore, homocysteine levels are elevated by over threefold in the retina of diabetic donors with documented retinopathy [22].

Continuous soaking of the retina in a hyperglycemic medium brings in many metabolic, molecular, functional, and structural changes including up—or downregulations of many metabolic pathways, thickening of the basement membranes, and leakage of retinal vasculature and impaired electrical activity of the retina in response to a light stimulus. Some of the major retinal metabolic abnormalities enabled by high glucose include activation of polyol and hexosamine pathways, protein kinase C, increased advanced glycation end product (AGE) formation, inflammation, and oxidative stress, and although high glucose itself can produce oxidative stress, other metabolic abnormalities initiated by hyperglycemia are also closely associated with increased oxidative stress [23,24].

## 2. Mitochondria

A diabetic environment increases oxidative stress in the majority of tissues including nerves and the retina, and both cellular and mitochondrial sources contribute to increased accumulation of retinal reactive oxygen species (ROSs). Cellular sources may include activation of NADPH oxidase 2 (Nox2) and metabolic abnormalities initiated by circulating high glucose including activation of polyol pathways and protein kinase C (PKC) and increased AGEs [23,24,25]. Mitochondria, the powerhouse of the cell, experiences a proton gradient when electrons are transferred through the electron transport chain (ETC) to synthesize adenosine triphosphate (ATP), and increased flux of electron donors into the ETC in a hyperglycemic milieu blocks electron transfer inside the complex III, increasing ROS levels [24,26,27].

Sustained hyperglycemia damages retinal mitochondria, and they show structural, functional, and genomic abnormalities. Mitochondria are swollen with partial crystolysis, their membranes are damaged allowing cytochrome c to leak out in the cytosol, and membrane potential is altered [24,25,26]. Mitochondria are very dynamic and, depending upon the energy demand, continuously undergo fusion and fission [28]. In diabetes, while mitochondrial fission is increased, their fusion is decreased. Activities of complex I and III of the ETC are inhibited, free radicals are elevated, and the enzyme responsible for scavenging mitochondrial free radicals, manganese superoxide dismutase (Sod2), is downregulated. In addition, mitochondrial DNA (mtDNA) is damaged, and its biogenesis is impaired [24,26,27]. Overexpression of *Sod2* in mice prevents diabetes-induced mitochondrial damage and protects them from developing diabetic retinopathy, strengthening the role of mitochondria in diabetic retinopathy [26].

How mitochondria are damaged in a hyperglycemic milieu is a complex process, which remains poorly elucidated. As mentioned above, ROSs are also constantly produced in the cytosol, and in diabetes Nox2 is activated. Experimental models have shown that Nox2 activation precedes mitochondrial damage and the development of diabetic retinopathy and have suggested a role of cytosolic ROSs in mitochondrial damage [29]. Sustained increase in cytosolic ROSs also activates matrix metalloproteinases -2 and -9 (MMP-2 and MMP-9), which are members of the extracellular matrix degrading protein family, with a critical role in vascular remodeling, angiogenesis, and apoptosis [30,31]. Activation of these cytosolic MMPs in the retina in diabetes is an early event that precedes mitochondrial damage. With the help of heat shock protein (Hsp70), these MMPs are transported inside the mitochondria, and by disrupting the gap junction protein connexin 43, they open up mitochondrial transition pores to allow cytochrome c to leak out. This activates the apoptotic machinery and accelerates capillary cell apoptosis, a phenomenon that precedes the pathology characteristic of diabetic retinopathy [30].

## 3. Homocysteine

As mentioned above, elevated levels of homocysteine are associated with an increased risk of diabetic retinopathy [8]. Homocysteine is biosynthesized from methionine by the removal of its terminal methionine group by S-adenosyl-methionine synthetase, forming *S*-adenosyl methionine (SAM). It can be re-methylated to L-methionine by methylenetetrahydrofolate reductase (MTHFR), or, with the help of cystathionine β-synthase (CBS), homocysteine can condense with serine to form cystathionine, which can be further converted to L-cysteine. Cysteine is an important amino acid for the biosynthesis of glutathione (GSH) and also a substrate for CBS and cystathionine-*γ* lyase (CSE) to form a gasotransmitter: hydrogen sulfide (H_2_S) [22,32,33]. Homocysteine is a by-product of transmethylation reactions, and proper functioning of methionine synthetase depends on vitamin B_12_ and folate as coenzymes [34], making folic acid and vitamin B_12_ the two vital regulators in its metabolism. Hyperhomocysteinemia could be the result of either impaired enzymatic function or a deficiency of folic acid/vitamin B_12_, or both [35].

High homocysteine, via the downregulation of CSE, reduces H_2_S in the microvasculature of diabetic mice [36]. H_2_S is considered an important signaling molecule (third gaseous) with important roles in a wide range of physiological and pathological conditions [37,38], and any imbalance between homocysteine and H_2_S increases oxidative stress, inflammation, and ischemic injury [39]. Our recent study has shown that in addition to significantly high homocysteine and low H_2_S, diabetic donors with established retinopathy have a compromised machinery to both transsulfurate and re-methylate homocysteine. They also have suboptimal CBS, MTHFR, and CSE [22], and due to decreased cysteine levels, biosynthesis of GSH, an intracellular antioxidant, is decreased, hampering the antioxidant capacity of the cell [40]. Homocysteine itself has an -SH group, which, in the presence of metal catalysts and molecular oxygen, is susceptible to oxidation [41]. Thus, homocysteine can exacerbate oxidative stress via several avenues, and our recent study has shown that addition of homocysteine in a high-glucose medium further increases ROS levels in the retinal endothelial cells, the cells that manifest histopathology characteristic of diabetic retinopathy [22]. Experimental models of diabetic retinopathy have shown an association between high homocysteine levels and various functional and structural abnormalities—it induces endothelial cell dysfunction, breakdown of the blood–retinal barrier, ischemia, endoplasmic stress, and inflammatory mediators [42]. In addition, polymorphisms of MTHFR, the enzyme essential for adding the methyl group to upstream folates, is considered as a risk factor for diabetic retinopathy [43]. 

## 4. Homocysteine and Mitochondrial Damage

Mitochondria have complex structure with double membranes and an intermembrane space between inner and outer membranes. To increase the surface area available for energy production, the inner membrane is also arranged into cristae and contains the enzymes of the tricarboxylic acid cycle. Retinal mitochondria are damaged in diabetes experiencing structural, functional, and genomic abnormalities. Complex I and III activities are inhibited, free radicals are elevated, and the enzyme responsible for scavenging free radicals in the mitochondria, Sod2, is downregulated. 

The mechanism by which diabetes damages the retinal mitochondria is complex and not clearly elucidated. Experimental models have shown that Nox2 activation precedes mitochondrial damage, and sustained increase in cytosolic ROSs damages the mitochondria [25,44]. In addition to Nox2, MMP-2 and MMP-9 are also upregulated in the retina in diabetes, and activation of these cytosolic MMPs is also an early event that precedes mitochondrial damage [30,31].

Our recent work has shown that addition of homocysteine in a hyperglycemic medium further increases ROS, activates MMP-9, and decreases the endogenous tissue inhibitor of MMP-9, Timp1. Furthermore, homocysteine also decreases interactions of Timp1 with MMP-9, which frees MMP-9 to become active [15]. The possible mechanism of MMP-9 activation by homocysteine appears to be via increased ROS production by homocysteine as MMP-9 has cysteine residues in the DNA-binding domain that are sensitive to oxidative stress [45,46]. In addition, lack of paraoxanase, with antioxidant properties, is considered to play a role in MMP-9-induced vascular endothelial impairment [47], and reduction in H_2_S in hyperhomocysteinemic conditions is shown to directly contribute to MMP activity [48]. As mentioned earlier, active MMPs damage the retinal mitochondria, which allows cytochrome c to leak into the cytosol and activate the apoptotic machinery.

To combat increased oxidative stress, the cell is equipped with many antioxidant defense systems consisting of both enzymatic and nonenzymatic components such as Sod2 and GSH [49,50]. Nuclear factor erythroid 2-related factor (Nrf2), a master regulator, regulates cellular oxidative stress by binding with an antioxidant-response elements, and initiating transcription of the genes for antioxidant enzymes including Sod2 and glutamate-cysteine ligase (GCLC), critical in GSH biosynthesis [50]. Under normal conditions, Nrf2 is kept in the cytoplasm by its intracellular inhibitor Kelch-like ECH-associated protein (Keap1), but when the cell experiences stress, Nrf2 moves inside the nucleus. In diabetes, Keap1 levels are elevated in the retina, hindering Nrf2 movement in the nucleus, and decreasing the transcriptional activity of Nrf2 [50,51]. Although homocysteine activates the Nrf2-mediated antioxidant response, which can protect cells from oxidative damage [52], chronic hyperhomocysteinemia downregulates Nrf2-responsive genes including *Sod2*, in retinal Muller cells, and Nrf2-lacking mice exposed to chronic high homocysteine have poor visual acuity, suggesting a role of homocysteine in the regulation of Nrf2-mediated antioxidant response and visual function [53,54]. Furthermore, reduction in GCLC decreases the biosynthesis of GSH, making the cell more vulnerable to oxidative damage [51,55]. Thus, decreased antioxidant defense, accompanied with increased ROS production, makes retinal mitochondria more vulnerable to oxidative stress in a hyperhomocysteinemic medium.

Mitochondria are also highly dynamic, and they adjust to the cellular demand by fusion, fission, mitophagy, and biogenesis. While fusion merges healthy mitochondria, mixing their DNA and other cellular components, fission removes the damaged mitochondrial fragments [56]. Biogenesis helps in restocking the mitochondrial pool and requires a coordinated effort of genes encoded by both nuclear and mitochondrial proteins. This delicate balance is essential for mitochondrial homeostasis and cell survival; impaired mitochondrial homeostasis is implicated in many diseases including diabetic retinopathy [57]. While mitochondrial fission is increased, their fusion is decreased, and mitophagy and biogenesis are impaired. Mitochondrial fission is mediated by GTPase dynamin related protein (Drp1 or Dnm1L), which acts in concert with outer mitochondrial membrane proteins, mitochondrial fission 1 protein and elongation factor 1. The fusion is regulated by outer and inner mitochondrial membrane proteins, mitofusins (Mfn1, Mfn2) and optic atrophy-1 (OPA1), respectively. These two processes occur almost simultaneously, and unhealthy/damaged/depolarized mitochondria are segregated for turnover by the autophagosomal-pathway mitophagy, a process closely associated with mitochondrial dynamics [58,59]. During mitophagy, microtubule associated protein, light chain 3 (LC3), is proteolytically cleaved by an ROS-dependent enzyme to LC3II [60]. Homocysteine also reduces mitochondrial respiration and decreases the activity of complex III of the ETC [61]. It impairs mitochondrial membrane permeability, releasing cytochrome c in the cytoplasm, and increasing caspase-3-mediated apoptosis in human umbilical vein endothelial cells [62]; in the pathogenies of diabetic retinopathy, capillary cell apoptosis precedes the development of diabetic retinopathy [63]. Homocysteine is also shown to increase endothelial cell damage in the mesenteric artery via a decreasing Mfn2/Drp1 ratio and an increasing mitophagy [64]. Ganglion cells of mice deficient in *CBS* have increased Fis1, Opa1, and fission, and their mitochondria are smaller in size compared to those of wildtype mice [65]. Diabetic donors with established retinopathy, in addition to increased retinal homocysteine levels, have an increased Drp1/Mfn2 ratio and increased levels of mitophagy markers [22]. This clearly suggests that homocysteine, in addition to damaging the mitochondrial structure, also impairs the removal of the damaged mitochondria.

Mitochondria contain their own DNA, which is circular and has only 16,500 base pairs. This small mtDNA encodes only 13 genes, compared to 20,000 different genes encoded by the nuclear DNA, but these 13 proteins are essential for the functioning of the electron transport chain [66,67], and cell survival becomes difficult even if one of these 13 proteins is compromised [68,69]. In diabetic retinopathy, mtDNA is damaged with increased mismatched base pairs, the transcription of mtDNA-encoded genes is decreased, and the electron transport chain is compromised [28,30,67,70]. Experimental data have shown that addition of homocysteine in a hyperglycemic milieu further increases mtDNA damage and compromising its transcriptional activity, decreasing transcription of the mtDNA-encoded genes essential for proper functioning of the ETC [70,71,72]. In addition to mtDNA damage, homocysteine also impairs mtDNA biogenesis via the decrease of mitochondrial transcription factor A [73], and in diabetic retinopathy, retinal mtDNA biogenesis is impaired [74]. Thus, high levels of homocysteine, especially in a hyperglycemic milieu, can exacerbate mtDNA damage and biogenesis, making it cumbersome for the mitochondria to function.

## 5. Homocysteine and Epigenetics

Biosynthesis of homocysteine from methionine, as stated above, forms SAM, and SAM is the primary methyl donor and a co-substrate in the DNA methylation process, emphasizing the role of homocysteine in epigenetic modifications. The importance of epigenetic mechanisms as the possible link between environmental stimuli and disease onset is now appreciated universally [75,76,77]. These epigenetic modifications, without altering the genomic sequence, activate or repress gene expression, and many genes implicated in the development of diabetic retinopathy undergo DNA methylation. In the pathogenesis of diabetic retinopathy, the retinal DNA methylation machinery is activated, and mtDNA itself is hypermethylated, resulting in impaired transcription of mtDNA-encoded genes and compromising of the ETC system. Furthermore, DNA at the promoters of *MMP-9* (associated with mitochondrial damage) and that of *Rac1* (Ras-related C3 botulinum toxin substrate, an obligatory protein for Nox2-activated cytosolic ROS production) also undergo dynamic DNA methylation, and the promoters of mtRas-related C3 botulinDNA biogenesis gene polymerase gamma (*POLG*) and that of mtDNA damage repair gene MutLH1 (*MLH1*) are hypermethylated. Thus, DNA methylation plays an integral role in the overall mitochondrial homeostasis in diabetic retinopathy. Our recent work has shown that homocysteine exacerbates glucose-mediated decreased transcription of mtDNA-encoded gene, cytochrome b [22]. Furthermore, data from both in vitro (human retinal endothelial cells in culture) and in vivo models (retinal microvasculature from streptozotocin-induced diabetic rats) models of diabetic retinopathy have shown that homocysteine supplementation further exacerbates hyperglycemia-induced activation of DNA methyl transferases (Dnmts), and hypermethylation of DNA at the promoter of *Timp1*, facilitating MMP-9-activated mitochondrial damage (unpublished data). Mitochondrial DNA itself is also hypermethylated in diabetes, and we cannot rule out the possibility that high homocysteine could cause its hypermethylation, further compromising the ETC system. Homocysteine metabolism itself is a complex process requiring various enzymes, and alterations in the DNA methylation of the genes encoding for these enzymes could alter their activity. Hypermethylated promoters of both *CBS* and *MTHFR* are found in the retinal microvasculature from human donors with diabetic retinopathy compared to the age-matched non diabetic donors, implying the role of DNA methylation in downregulation of *CBS* and *MTHFR* [22]. We recognize that although the effect of homocysteine in regulating glucose-induced DNA methylation is still in its incipient stages, the availability of the methyl donor during homocysteine biosynthesis also provides an additional opportunity for the activation of DNA methylation and altering the transcription of many genes associated with maintaining mitochondrial homeostasis.

Different epigenetic mechanisms are also inter-related; e.g., DNA methylation can alter histone modifications and microRNAs can affect DNA methylation or histone modifications [78], and the role of high homocysteine in epigenetic modifications, other than DNA methylation, cannot be ruled out. In fact, a recent study has shown that high homocysteine increases histone deacetylase activity and alters many miRNAs implicated in the activation of Dnmts, inflammation, oxidative stress, endoplasmic reticulum stress, and angiogenesis [79].

## 6. Strategies to Regulate Homocysteine and Protect Mitochondrial Damage

It is well established that the development of diabetic retinopathy, and other complications of diabetes, can be prevented/inhibited by maintaining a healthy lifestyle and maintaining good glycemic control [80]. However, maintenance of tight glycemic control for a life-long disease can be challenging, or at times, not feasible, for many patients. In addition, as detailed above, high homocysteine levels further make a diabetic patient prone to developing complications including retinopathy, and strategies targeting increased homocysteine levels could provide patients some relief from the development/worsening of this devastating blinding disease.

Since folic acid and vitamin B_12_ regulate homocysteine metabolism, this makes dietary folic acid/vitamins B_6_/B_12_ the most important modifiable factors in maintaining homocysteine levels. However, trials with folic acid supplementation have been inconclusive, oral folic acid supplementation for three months in patients with hyperhomocysteinemia has not been very successful in bringing their homocysteine levels to the normal range [81], and folic acid, vitamin B_6_, vitamin B_12_ have produced little or no effect on cardiovascular mortality [82]. Furthermore, a clinical study employing patients with folic acid and vitamin B_12_ in the normal reference range has shown a close correlation between increased serum homocysteine and severity of diabetic retinopathy [7], raising concerns about the beneficial effects of folic acid. There still remains a possibility that consuming food rich in folic acid could be beneficial; the major source of folic acid is green vegetables, fruits, and bean, and the consumption of less fatty meat, more fish, and fruit or vegetables should help maintain good homocysteine levels, and possibility retard diabetic retinopathy.

In diabetes, while plasma homocysteine levels are elevated, H_2_S levels and CSE activity are decreased [8,83], therefore, another viable strategy could be, instead of focusing on lowering homocysteine alone, targeting the homocysteine–H_2_S metabolic balance. Metabolic imbalance of homocysteine–H_2_S is implicated in many pathological conditions including oxidative stress, inflammation, and ischemia/reperfusion injury [39]. H_2_S protects brain endothelial cells from methionine-induced oxidative stress, and decline in its levels contributes to endothelial dysfunction in cerebral ischemia/reperfusion [84,85]. Both human and animal models have demonstrated the role of H_2_S in vascular inflammation and cardiomyopathy [86]. Our ongoing work using a rodent model of diabetic retinopathy has shown that administration of a slow-releasing H_2_S donor, GYY4137, in addition to restoring H_2_S and homocysteine levels, prevents the development of retinopathy in diabetic mice (unpublished), strengthening the importance of maintaining a normal homocysteine–H_2_S balance. In addition, there are many drugs available in the market containing sulfur moieties that release H_2_S ex vivo. These drugs may release H_2_S in vivo, and the sulfur moieties could function as an H_2_S donor affecting endogenous H_2_S metabolism; however, further research is needed to clarify the pharmacotherapeutic profile of parent drugs [87]. Efforts are being made in many laboratories to develop better H_2_S-releasing/-stimulating compounds [88,89]. With recent advances in our knowledge and technical advances, the field has made great progress. The future for developing controllable H_2_S donors and different H_2_S-releasing mechanisms looks optimistic.

## 7. Conclusions

**Figure 1:**
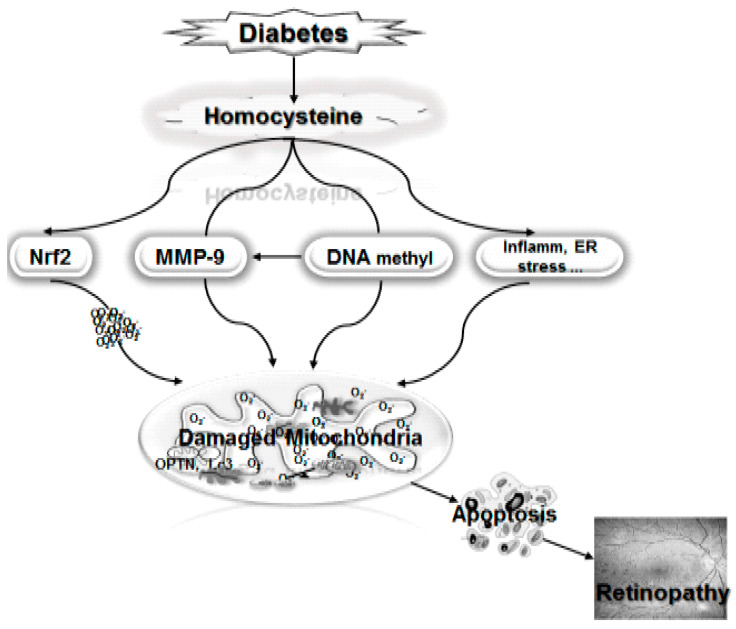
Homocysteine, by activating matrix metalloproteinase-9 (MMP-9) facilitates the damage of mitochondria, and by inhibiting the transcriptional activity of nuclear factor erythroid 2-related factor (Nrf2), damages the defense system. By supplying S-adenosyl methionine (SAM), it helps in the DNA methylation process and the hypermethylation of mitochondrial DNA (mtDNA) and that of *POLG* and *MLH1* and impairs the electron transport chain by interfering with the transcription, biogenesis, and repair of damaged mtDNA. The vicious cycle of free radicals continues to self-propagate. Homocysteine can also increase inflammation and endoplasmic reticulum (ER) stress and other pathways implicated in mitochondrial damage.

Thus, as shown in Figure 1, high homocysteine activates various pathways associated with mitochondrial damage and diabetic retinopathy, the activation of MMP-9 by homocysteine accelerates mitochondrial damage, and the inhibition of the transcriptional activity of Nrf2 compromises the defense system’s ability to overcome the increased oxidative stress. It can also activate inflammation and endoplasmic reticulum (ER) stress, and many other pathways, increasing mitochondrial damage. In addition, the formation of SAM during homocysteine biosynthesis from methionine, further activates Dnmts, altering the DNA methylation status of many genes implicated in mitochondrial homeostasis including *MMP-9*, *POLG*, *Rac1*, and *mtDNA* itself. Thus, high homocysteine finds many avenues to damage the mitochondria, accelerating capillary cell apoptosis and the development of diabetic retinopathy. Inhibition of hyperhomocysteinemia in diabetic patients could help them prevent the development of this sight-threatening disease.
